# Medical and Ethical Concerns Regarding Pacemaker Implantation in a Patient with Substance Use Disorder

**DOI:** 10.7759/cureus.3027

**Published:** 2018-07-22

**Authors:** Srilekha Sridhara, Patricia A Mayer

**Affiliations:** 1 Internal Medicine, Banner Baywood Medical Center, Mesa, USA; 2 Palliative Care and Medical Ethics, Banner Health, Mesa, USA

**Keywords:** intravenous drug use, pacemaker, drug rehabilitation, ethics

## Abstract

Medical and ethical dilemmas surrounding endocarditis and cardiac valve replacements related to intravenous drug use have been described often. Less well-described are dilemmas associated with pacemaker implantation in such patients. We describe a patient with a substance use disorder for whom a pacemaker was medically indicated.

## Introduction

Cardiac implantable electronic device infections in substance use disorder patients are less well described in medical literature. We discuss treatment of a patient with a substance use disorder requiring a pacemaker implantation, informed consent process, risk factors for device infections, and management options.

## Case presentation

A 47-year-old female with an active substance use disorder involving intravenous (IV) heroin, amphetamines, benzodiazepines, and a known history of asthma and chronic obstructive pulmonary disease (COPD) was found cyanotic at home in a prone position. She was hypoxemic on room air with oxygen saturation of 50% but emergency medical services (EMS) were unable to intubate her so she arrived at the emergency room (ER) on 100% supplemental oxygen through a bag and mask ventilation. Once in the ER, she was intubated and treated with albuterol and ipratropium nebulizations.

Physical exam revealed bilateral rhonchi, a pulse of 89 beats per minute, and a blood pressure of 120/58. Laboratory values included a white cell count of 12,000 k/mm^3^, hemoglobin 13 g/dl, d-dimer 6,481 ng/ml, blood urea nitrogen 90 mg/dl, creatinine 0.96 mg/dl, lactic acid 2.5 mmol/L, and an arterial blood gas with a pH of 7.29, partial pressure of carbon dioxide of 46 mm Hg, partial pressure of oxygen of 110 mm Hg on 35% fractional inspired oxygen, and a peak end-expiratory pressure of 5. Her urine drug screen was positive for methamphetamines, benzodiazepines, and opiates, and a chest radiograph showed a right lung infiltrate. Blood cultures were drawn which showed no growth.

She was diagnosed with acute hypoxic and hypercapnic respiratory failure secondary to asthma exacerbation with possible aspiration pneumonia. She was treated with intravenous steroids and antibiotics and was placed on a substance withdrawal protocol with lorazepam, clonidine, Robaxin, and as needed Seroquel. 

The patient self-extubated 24 hours after admission and became agitated and tachycardic. She then had a brief run of high-grade (advanced) atrioventricular block (AVB) with a 9:1 conduction abnormality and 2:1 AVB.

Subsequent electrocardiography (EKG) showed a sinus rhythm with no conduction abnormalities. Echocardiography revealed no regional wall motion abnormalities, valvular dysfunction, or vegetations. She remained hemodynamically stable. Electrophysiology was consulted and recommended a permanent pacemaker placement. Review of her electronic medical records stated the risks of pacemaker placement were discussed with the patient and her family and they consented. Details of the conversation were not recorded. The pacemaker was placed with anesthesia support without complications, and periprocedural antibiotic coverage was provided.

Information regarding drug rehabilitation was provided by the psychiatry team, along with recommended follow-up with an addiction specialist as an outpatient. This information was provided both before and after the procedure. The patient initially agreed to an inpatient drug rehabilitation program but later refused and was discharged home. At follow-up two weeks post-pacemaker placement, she was doing well without substance abuse relapse.

## Discussion

Our patient had no previous known cardiac history. The syncopal spell prior to admission could have been caused by respiratory distress, substance abuse, an episode of the same heart block observed in the hospital, or something unknown.

Patients with a high-grade atrioventricular block (AVB) not treated with a pacemaker have about 31% higher rates of death than those treated with one as AVB can be progressive and result in sudden cardiac death [1]. This diagnosis is considered a Class I recommendation for permanent pacemaker implantation [2].

However, any implanted foreign body is highly susceptible to infection, particularly in a patient who is known to have an IV substance use disorder. Therefore, her chance of infection is quite high if she relapses.

There was an indication for pacemaker placement in our patient because of syncope and high-grade AVB despite considerable concern for pacemaker infection, as well as valve endocarditis, if she relapsed. Although she had not relapsed at two weeks, her rejection of rehabilitation is not promising for long-term abstinence.

The baseline rate of cardiac implantable electronic device (CIED) infection ranges from 0.8 to 5.7% [3]. In addition, the mortality of intravenous (IV) drug use-related endocarditis increases significantly in patients with CIED [4].

CIED infections can be pocket or systemic infections. In the former, infection is in either the generator pocket or the subcutaneous component of the leads. Perioperative contamination of the pacemaker site with skin flora is the most common source of generator pocket infection, although lead erosion can cause a local infection. Lead erosion usually happens at a time remote from the CIED placement. Coagulase-negative Staphylococcus aureus is the most common cause of device pocket-related infection, is less virulent compared to Staphylococcus aureus, and has fewer systemic symptoms. In the latter, the involvement of transvenous leads or heart valves manifesting as endocarditis remains an important form of presentation. Epicardial lead involvement can cause pericarditis or mediastinitis.

To diagnose CIED infection, two sets of blood cultures, device pocket swabs for gram stain, culture, and tissue culture at the time of device removal, and lead tip or entire lead culture are required. A transesophageal echocardiogram is a useful diagnostic modality for confirmation of endocarditis, valvular regurgitation, perivalvular abscess, and for the guidance of antibiotic therapy and possible reimplantation timing.

Other modalities utilized are positron emission tomography using 18F-labeled fluorodeoxyglucose computed tomography (CT) (particularly in bacteremic patients with non-diagnostic echocardiograms) and single-photon emission CT with CT scintigraphy with radiolabeled white blood cells for CIED-associated endocarditis and septic embolism [5]. Depending on the extent of the infection and microbiology, antibiotics and device removal are treatment modalities. Re-implantation of the pacemaker after explantation will require sterile blood cultures if the indication for pacemaker placement still exists [3, 6]. Re-implantation would be complicated in a patient with a substance use disorder with continued active use of IV drugs.

Management options for pacemaker-related infections include epicardial lead reimplantation before device extraction or temporary pacing with delayed endocardial reimplantation. Perrin et al. reported the latter option had a reduced risk of late endocarditis, though there was no difference in long-term mortality between the two strategies [7]. Amarousi et al. reported both strategies offered an excellent success rate and low risk of complications with lower hospital length of stays for surgical epicardial lead placement [8]. There was, however, the risk of increased device infection with epicardial leads [9]. Leadless pacemakers and low lateral thoracic site pacemaker placement are viable options for patients after device extraction [10-11]. Survival in CIED infection patients remained poor even after effective transvenous lead extraction (TLE), and clinical risk factors for infection were also predictive of post-TLE mortality [12]. However, these studies do not specifically address patients with substance use disorder as the cause of CIED infections.

We note the cost of pacemaker placement varies across the United States and ranges between $20,000 and $90,000 [13]. The cost of removal, antibiotics, and reinsertion is much higher. The cost of addiction rehabilitation is significantly less but requires societal buy-in and an available program; such programs are often not covered by insurance and thus may be unaffordable to patients in need. A needle exchange program for patients who use intravenous drugs would offer a way to avoid infection. Needle exchange programs as a component of harm reduction programs for this purpose in the Netherlands have been successful [14]. These also ought to decrease the risk of right-sided endocarditis and implant infections.

Clinicians are commonly faced with ethical dilemmas when caring for patients with a substance use disorder who develop infective endocarditis, particularly if they develop florid heart failure due to valvular dysfunction. Continued drug use after valve replacement may lead to prosthetic valve endocarditis and outcomes are largely determined by microbiology [15]. In such cases, re-operation might not be possible due to altered cardiac anatomy and patient factors, such as continued substance use disorder or poor social/family support, conferring the risk for recurrent endocarditis [15]. In light of the growing opioid epidemic, much is discussed about treatment options and ethical aspects of IV drug use and endocarditis [16]. Although the recurrent infection risk for patients with IV substance use disorder requiring pacemakers is similar to those requiring valve replacement, literature for such cases is scarce.

The medical options for patients who need pacemakers are different than for patients with infectious endocarditis. Some patients with endocarditis can be treated with long-term IV antibiotics. However, there is no available alternative to a pacemaker in a patient with a high-grade atrioventricular (AV) block. Pacemaker placement remains standard therapy unless a patient refuses.  

However, because of the risk of infection with IV drug use, a tailored discussion and informed consent were warranted regarding implantation. The medical record indicates risks were discussed. This should have included specific risks and benefits (as well as acknowledgment of unknowns) regarding this procedure in light of her specific history. For instance, the infection rate would be much higher for her than in a patient who did not use IV drugs. Unfortunately, alternative treatments are not readily available. It seems unlikely that this patient would have left the hospital without a pacemaker unless she refused it. It may have been useful to have an addiction specialist present for the conversation and a clear plan for inpatient rehabilitation finalized (along with the financial resources required to make the plan a reality) prior to permanent implantation. 

Informed consent must always be tailored to an individual’s unique presentation and medical history. Her consent ideally would have included such information. In addition, drug use cessation with a rehabilitation program should be a cornerstone of treatment and could be facilitated by the cardiologist, primary care physician, and addiction specialist, along with case management, social work, and possibly finance.

It is worth noting that treatment of an infected pacemaker is generally simpler than treatment of an infected valve. The pacemaker can be removed and not replaced until the infection has been eradicated, although this would require continuous monitoring.

Risk factors associated with a pacemaker infection, in general, are diabetes mellitus, chronic obstructive pulmonary disease, malignancy, end-stage renal disease, local skin infection, post-procedural hematoma, lack of antibiotic prophylaxis, and device replacement or revision. We now add substance use disorder as a risk factor for CIED infection in reference to a case report of a similar patient with a pacemaker who developed a pacemaker lead endocarditis [17].

## Conclusions

This case illustrates IV drug usage as a risk factor for CIED infections and highlights the need for a careful, individualized, and comprehensive consent process and treatment program when considering permanent pacemakers in such patients.
